# Trends in incidence of lung cancer in Croatia from 2001 to 2013: gender and regional differences

**DOI:** 10.3325/cmj.2017.58.358

**Published:** 2017-10

**Authors:** Katarina-Josipa Siroglavić, Marina Polić Vižintin, Ingrid Tripković, Mario Šekerija, Suzana Kukulj

**Affiliations:** 1 Department of Public Health, Andrija Štampar Teaching Institute of Public Health, Zagreb, Croatia; 2 Department of Public Health, Teaching Institute of Public Health for Split – Dalmatia County, Split, Croatia; 3 Division for Epidemiology and Prevention of Noncommunicable Chronic Diseases, Croatian Institute of Public Health, Zagreb, Croatia; 4 Department of Medical Statistics, Epidemiology, and Medical Informatics, Andrija Štampar School of Public Health, University of Zagreb School of Medicine, Zagreb, Croatia; 5 Department for Mediastinal Tumors, Clinic for Lung Diseases Jordanovac, University Hospital Center Zagreb, Zagreb, Croatia

## Abstract

**Aim:**

To provide an overview of the lung cancer incidence trends in the City of Zagreb (Zagreb), Split-Dalmatia County (SDC), and Croatia in the period from 2001 to 2013.

**Method:**

Incidence data were obtained from the Croatian National Cancer Registry. For calculating incidence rates per 100 000 population, we used population estimates for the period 2001-2013 from the Croatian Bureau of Statistics. Age-standardized rates of lung cancer incidence were calculated by the direct standardization method using the European Standard Population. To describe incidence trends, we used joinpoint regression analysis.

**Results:**

Joinpoint analysis showed a statistically significant decrease in lung cancer incidence in men in all regions, with an annual percentage change (APC) of -2.2% for Croatia, 1.9% for Zagreb, and -2.0% for SDC. In women, joinpoint analysis showed a statistically significant increase in the incidence for Croatia, with APC of 1.4%, a statistically significant increase of 1.0% for Zagreb, and no significant change in trend for SDC. In both genders, joinpoint analysis showed a significant decrease in age-standardized incidence rates of lung cancer, with APC of -1.3% for Croatia, -1.1% for Zagreb, and -1.6% for SDC.

**Conclusion:**

There was an increase in female lung cancer incidence rate and a decrease in male lung cancer incidence rate in Croatia in 2001-20013 period, with similar patterns observed in all the investigated regions. These results highlight the importance of smoking prevention and cessation policies, especially among women and young people.

Lung cancer is the most common cancer in the world, with 1.8 million new cases diagnosed in 2012 and accounting for 12.9% of total cancer incidence (1). In terms of mortality, 1.6 million deaths were caused by lung cancer in 2012, accounting for 19.4% of total cancer deaths (1). Given the great public health burden imposed by lung cancer and the large differences in the incidence of lung cancer in various areas or within different time periods due to changing population structure and varying levels of smoking exposure, it is imperative to continuously monitor and assess data on epidemiological indicators. In 2012, Croatia was among 20 countries with the highest incidence of lung cancer in the world: the incidence rate (age-standardized to standard world population; ASR-W) was 34.3 per 100 000 persons (1). According to the most recent data available from the Croatian National Cancer Registry, in 2013, lung cancer was the most common cancer in men with crude incidence rate of 99.9/100 000 persons and the third most common cancer in women with crude incidence rate of 33.2/100 000 persons (2). Crude mortality rate in 2013 was 101.8/100 000 persons for men and 32.3/100 000 for women (2).

Lung cancer incidence largely mirrors smoking prevalence, with a latency period of several decades (3). Tobacco smoking and exposure to environmental tobacco smoke is a proven cause of lung cancer (4). Cigarette smoking accounts for approximately 80% of lung cancer cases in men and 50% of lung cancer cases in women worldwide (5). Lung cancer is also caused by secondhand tobacco smoke, with estimated 21 400 lung cancer deaths in non-smokers annually (6). Other risk factors for lung cancer include indoor air pollution because of unventilated combustion of coal in households for heating and cooking; outdoor air pollution; occupational exposure to hazardous chemicals, such as in coal gasification and aluminum production; exposure to radiation from indoor radon released from soil and building materials, and exposure to asbestos, silica dust, and several elements, including arsenic (7). Only a small number of patients diagnosed and treated for lung cancer have never smoked tobacco according to a study conducted on 212 newly diagnosed lung cancer patients at a tertiary level hospital in Zagreb (8).

Lung cancer is the leading cause of the years of life lost because of cancer and is associated with the highest economic burden relative to other cancer types. Thus, it represents a major public health problem. However, according to available data, the level of world research output on lung cancer lags significantly behind research on other malignancies (9). To achieve improved outcomes of lung cancer, more studies and research are necessary. We wanted to emphasize the importance of this public health issue and elucidate recent changes in the burden of lung cancer in Croatia.

Specifically, our aim was to assess trends of lung cancer incidence in two Croatian counties with two largest cities in Croatia: the City of Zagreb (Zagreb) in the continental part of Croatia and Split-Dalmatia County (SDC), with Split as its capital, in coastal part of Croatia in the period from 2001 to 2013 and trends in the entire country, emphasizing possible regional and gender-specific differences.

## MATERIAL AND METHODS

### Data sources

The incidence data for the period 2001-2013 were obtained from the Croatian National Cancer Registry (CNCR). Lung cancer was defined according to the International Classification of Diseases (ICD), 10th Revision, codes C33 and C34, including malignant neoplasms of the trachea, bronchus, and lung. CNCR, founded in 1959, covers the whole Croatian population of approximately 4.2 million people and relies on mandatory cancer notifications from primary and secondary health care sources, copies of pathological findings from pathology departments, and death certificates from the Croatian Bureau of Statistics. Data from the CNCR are included in the Cancer Incidence in Five Continents publications. For calculating incidence rates per 100 000 population, we used population estimates for years 2001-2013 from the Croatian Bureau of Statistics. For age-standardized rates (ASR), we used the European standard population (10).

### Statistical analysis

Age-standardized rates of lung cancer incidence (ASR-E) in Zagreb, SDC, and Croatia were calculated by the direct standardization method using the European Standard Population from year 1976 as a reference (10).

To describe incidence and mortality time trends, we carried out joinpoint regression analysis of age-standardized rates using Joinpoint Regression Program, Version 4.5.0.0, from 2017. This approach takes rates of events – in this case, age-standardized cancer incidence rates – and tries to fit the simplest model possible, starting with the model with zero joinpoints (11). Using Monte Carlo Permutation method, this approach tests whether adding additional joinpoints (up to a previously defined maximum) is statistically significant. It allows the user to see if the change in trend is statistically significant. For the period between two joinpoints (or the beginning and the end of series), a log-linear model with annual percent change (APC) is calculated as a trend measure, with its corresponding confidence intervals (CI).

This analysis included logarithmic transformation of the rates, standard error for heteroscedasticity, maximum number of two joinpoints, a minimum of four years between two joinpoints, and a minimum of three years between a joinpoint and beginning or the end of the series. A permutation test was used with statistical significance set at *P* < 0.05.

In describing trends, the terms “significant increase” or “significant decrease” signify that the slope of the trend was statistically significant (*P* < 0.05), with CI not including zero, against a null-hypothesis of APC = 0. All statistical tests were two-sided.

## RESULTS

### Regional differences

The overall number of diagnosed lung cancer cases in Croatia decreased from 2428 in 2001 to 2051 in 2013, which was a decline of 15.5%. The number of diagnosed lung cancer cases in the City of Zagreb decreased from 397 to 360 in the same time period, representing a decline of -9.3%. In SDC, there was a small increase in the number of lung cancer cases from 202 in 2001 to 207 in 2013, ie, a +2.5% increase (Table 1). Age-standardized rates declined in all regions.

**Table 1:** Lung cancer incidence in Zagreb, Split-Dalmatia County (SDC), and Croatia in the period 2001-2013

	Incidence								
	Zagreb			Split-Dalmatia County			Croatia		
Year	No. of cases	ASR*	IR^‡^	No. of cases	ASR*	IR^‡^	No. of cases	ASR*	IR^‡^
Men									
2001	397	97.9	109.8	202	89.6	95.6	2428	103.6	117.5
2002	421	103.3	116.7	190	82.8	89.1	2358	99.2	114.1
2003	343	83.9	95.0	199	85.0	92.7	2224	92.3	107.6
2004	337	80.7	93.1	228	93.6	105.5	2143	88.1	103.6
2005	405	95.8	111.6	258	105.9	118.3	2329	94.7	112.4
2006	368	85.1	101.2	215	86.7	97.9	2167	87.9	104.5
2007	355	82.0	97.3	242	93.8	109.8	2219	88.5	106.9
2008	323	72.3	88.2	215	82.8	97.3	2040	80.3	98.2
2009	439	97.7	119.5	196	72.9	88.6	2242	87.3	108.0
2010	284	62.4	77.1	200	76.0	90.4	2033	78.3	98.2
2011	397	87.5	107.4	215	79.4	97.2	2175	83.3	105.4
2012	349	75.2	94.1	208	73.2	94.0	2088	78.0	101.4
2013	360	77.3	96.8	207	73.6	93.5	2051	76.6	99.9
Women									
2001	142	25.8	34.7	60	23.0	26.8	548	18.2	24.5
2002	141	24.6	34.3	57	20.3	25.3	593	19.4	26.5
2003	147	25.4	35.7	55	18.9	24.2	555	17.5	24.8
2004	142	26.0	34.4	54	17.8	23.6	587	18.5	26.3
2005	151	26.6	36.5	81	28.2	35.1	646	20.6	28.9
2006	153	26.1	36.9	50	17.0	21.6	655	21.0	29.3
2007	124	21.5	29.8	70	22.0	30.1	572	17.8	25.6
2008	124	19.3	29.7	57	16.7	24.5	519	15.7	23.2
2009	183	30.0	43.8	53	16.6	22.7	706	21.4	31.7
2010	124	20.1	29.5	63	19.4	27.0	660	19.8	29.7
2011	199	32.1	47.3	73	21.9	31.3	772	22.8	34.8
2012	164	26.8	38.8	66	19.0	28.3	691	20.7	31.3
2013	174	29.3	41.1	70	20.4	30.0	730	21.8	33.2

ASR* - age standardized rate (using European standard population) per 100 000 population

IR^‡^ - crude incidence rate per 100 000 persons per year

### Men

Lung cancer ASR-E in men decreased from 97.9/100 000 in 2001 to 77.3/100 000 in 2013 in Zagreb, from 89.6/100 000 to 73.6/100 000 in SDC, and from 103.6/100 000 to 76.6/100 000 in Croatia. A joinpoint analysis showed a significant decrease in ASR-E in all analyzed regions, with the highest APC of -2.2% (95% CI, from -2.4% to -2.0%) for Croatia, followed by APC of -2.0% (95% CI, from -2.4% to -1.5%) for SDC, and APC of -1.9% (95% CI, from -2.6% to -1.3%) for Zagreb (Table 2)

**Table 2:** Joinpoint analysis of age-standardized rates of lung cancer incidence in men and women of all ages in Zagreb, Split-Dalmatia County, and Croatia, 2001-2013*

Region	Sex	APC	95% CI
ZG	M	-1.9	-2.6 - -1.3
ZG	F	1.0	0.3 - 1.8
ZG	M/F	-1.1	-1.7 - -0.5
SDC	M	-2.0	-2.4 - -1.5
SDC	F	-0.8	-1.6 - 0.0
SDC	M/F	-1.6	-2.1 - -1.1
Cro	M^¶^	-2.2	-2.4 - -2.0
Cro	F	1.4	1.0 - 1.9
Cro	M/F	-1.3	-1.5 - -1.0

*Abbreviations: APC - annual percentage change, CI –confidence interval, ZG – Zagreb, M – male, F – female, SDC – Split-Dalmatia County, Cro – Croatia.

### Women

In women, there was an increase in the number of new lung cancer cases in 2013 in comparison with 2001 for Croatia (730/548, +33.2%), Zagreb (174/142, +22.5%), and SDC (70/60, +16.7%) (Table 1). Lung cancer ASR-E in women in Croatia increased from 18.2/100 000 in 2001 to 21.8/100 000 in 2013 and in women in Zagreb, it increased from 25.8/100 000 in 2001 to 29.3/100 000 in 2013. In women in SDC, it decreased from 23.0/100 000 to 20.4/100 000 in the same time period.

Joinpoint analysis showed a significant increase in the ASR-E of lung cancer in women for Croatia (Table 2). There was also a significant increase in the ASR-E of lung cancer in women Zagreb, but no statistically significant change in trend was found for women in SDC.

In terms of both sexes, joinpoint analysis showed a significant decrease in ASR-E of lung cancer for entire Croatia, the city of Zagreb, and SDC (Table 2).

## DISCUSSION

This is the first study to simultaneously assess trends in incidence rates of lung cancer in different regions of Croatia. It shows that there is an overall decreasing trend in lung cancer incidence in Croatia, with still opposing trends in men and women, as discussed in a study by Janković et al (12). However, according to GLOBOCAN 2012, Croatia is still among the European countries with the highest lung cancer incidence and mortality rate, especially in men (2). Quality of lung cancer data collection and reporting varies between countries due to the variation in the existence and quality of cancer registries, differences in the percentage of cases reported only by death certificates (DCO cases) and challenges in determining primary cancer site in low-resource settings.

Our results show significant decrease in incidence rate for men in Zagreb, SDC, and overall Croatia and significant increase for women in Zagreb and Croatia. In our study, incidence rates were considerably lower among women, which is associated with a lower smoking prevalence in women (13), but our study also showed diverging time trends of lung cancer incidence in men and women. A previous study has suggested that a higher proportion of women than men in Croatia are starting to smoke (14), so there is little probability that these worrying trends in women are likely to reverse in the years to come, unless comprehensive preventive measures are implemented.

It is estimated that tobacco smoke contains around 4800 compounds of which about 100 are carcinogenic, mutagenic or have tumor promoter properties (14). Recent data indicate that more than one-quarter of adult inhabitants of Croatia are everyday smokers, which presents a great public health problem (15). The Croatian Adult Health Survey (CAHS) performed in 2003 suggested regional differences in smoking prevalence and a higher prevalence in men. However, regional difference in smoking prevalence between the City of Zagreb and Adriatic region was not significant. The greatest gender differences were recorded in the City of Zagreb, with a strong women predominance pattern (13). This could be related to the modifying effects of the level of urbanization associated with the weakening the conservative and traditional opinions and lifestyle patterns.

By ratifying the World Health Organization’s Framework Convention for Tobacco Control (WHO FCTC), Croatia integrated European tobacco bans into Croatian Act on the restriction of the use of tobacco products: restricted areas for smokers, limitation of tar and nicotine levels, and advertising forbidden through the country. However, there is a problem with its implementation, meaning that the smoking is still allowed in most of the bars and cafes (16). Better law enforcement would certainly contribute to further reducing the prevalence of smoking.

Croatia should look up to countries who have the most comprehensive tobacco control measures (eg, the United Kingdom), which include high prices, comprehensive smoke free legislation, comprehensive advertising bans, and pictorial health warnings (17). Raising taxes to increase tobacco product prices is the most effective and cost-efficient means of reducing tobacco use and encouraging users to quit, but it is one of the least used tobacco control measures (18). Croatia should also have better programs to help people who want to quit smoking framed within an integrated strategy or a national plan.

According to the Association of European Cancer Leagues (17), future steps in tobacco control that could be applicable for Croatia include implementation of a comprehensive tobacco control policy in its tobacco control program, which is an obligation under Article 4 of the WHO FCTC, with the introduction of various measures defined in the convention. Other possible measures include investing more in public awareness campaigns, which should be organized annually to ensure that they reach more patients at the right time, establishing better control of tobacco sales to minors, and consider and debate about implementing screening methods, such as low dose computed tomography (LDCT) given that some studies show potential benefits of this screening method (19). There should also be a better control of tobacco sales to minors. Those measures have been proven to be effective to reduce smoking.

Our study showed an increase in female lung cancer incidence rate and a decrease in male lung cancer incidence rate in Croatia. Despite that, Croatia is still among the European countries with the highest lung cancer incidence. Given the high incidence and mortality rate of lung cancer in Croatia, introducing a comprehensive tobacco control program, especially among young people and women, is of utmost importance.

Possible limitations of our study include uneven and delayed reporting of lung cancer cases in Croatia in some years that may reflect in larger rate variations than expected. Short time frame of the study is also one of the possible limitations.

Different measures likely have synergistic effects, and the consensus is that a comprehensive, multi-stakeholder approach is the most effective means of reducing tobacco consumption. The differences in trend between two largest Croatian counties, and differences in comparison of both overall incidence and incidence trends to overall data for Croatia, warrant further studies extended to other Croatian areas, in order to elucidate factors that might contribute to these differences and to tailor preventive programs accordingly.
